# Antiviral Activity of *Isatis indigotica* Extract and Its Derived Indirubin against Japanese Encephalitis Virus

**DOI:** 10.1155/2012/925830

**Published:** 2012-07-17

**Authors:** Shu-Jen Chang, Yi-Chih Chang, Kai-Zen Lu, Yi-Yun Tsou, Cheng-Wen Lin

**Affiliations:** ^1^School of Pharmacy, China Medical University, Taichung 404, Taiwan; ^2^Department of Medical Laboratory Science and Biotechnology, China Medical University, Taichung 404, Taiwan; ^3^Department of Biotechnology, College of Health Science, Asia University, Wufeng, Taichung 413, Taiwan

## Abstract

*Isatis indigotica* is widely used in Chinese Traditional Medicine for clinical treatment of virus infection, tumor, and inflammation, yet its antiviral activities remain unclear. This study probed antiviral activity of *I. indigotica* extract and its marker compounds against Japanese encephalitis virus (JEV). *I. indigotica* methanol extract, indigo, and indirubin proved less cytotoxic than other components, showing inhibitory effect (concentration-dependent) on JEV replication *in vitro*. Time-of-addition experiments proved the extract, indigo, and indirubin with potent antiviral effect by pretreatment (before infection) or simultaneous treatment (during infection), but not posttreatment (after entry). Antiviral action of these agents showed correlation with blocking virus attachment and exhibited potent virucidal activity. In particular, indirubin had strong protective ability in a mouse model with lethal JEV challenge. The study could yield anti-JEV agents.

## 1. Introduction

*Isatis indigotica* is an herb distributed widely in China and traditionally used in clinical treatment of viral diseases like influenza, hepatitis, and encephalitis [1, 2]. Accumulated experimental evidence indicates it and related components as associated with antiviral activity against influenza A, SARS-coronavirus, foot-and-mouth disease, rabies, and human immunodeficiency virus type 1 (HIV-1), among others [1, 3–9]. Among natural compounds identified from *I. indigotica*—for example, indican, isatin, indirubin, and indigotin [9]—indirubin exhibits multiple immunomodulatory and antiviral effects [5, 8].

Japanese encephalitis virus (JEV) belongs to genus *Flavivirus* of the Flaviviridae family, an arthropod-borne microorganism [10]. Vaccines against it are currently available and effective, yet viruses' zoonotic characteristic and occasional infections cause JEV to rank as a leading cause of high morbidity and mortality rate in Southeast Asia and the Western Pacific region [11]. 30–50% of JE patients develop permanent neuropsychiatric sequelae, while 20–30% of JE cases result in death [12]. Extensive study to develop new therapeutic strategy may be needed. This study rated inhibitory effect of ethyl acetate, methanol, and water extracts of *I. indigotica*, along with its related natural compounds, on JEV replication. We proved that pretreatment of *I. indigotica* extracts, indigo, and indirubin greatly inhibit JEV replication *in vitro*. These agents blocked JEV attachment, which correlated with a potent virucidal activity.

## 2. Materials and Methods

### 2.1. Viruses and Cells

JEV strain T1P1 was used as previously described [13], vero cells for JEV amplification maintained in Dulbecco's modified Eagle's medium (DMEM), as well as BHK-21 cells used to determine JEV plaques grown in minimum essential medium (MEM) supplemented with 10% fetal bovine serum (FBS). Human promonocytic HL-CZ cells cultured in RPMI-1640 medium served to determine JEV yield *in vitro*.

### 2.2. *I. indigotica* Extracts and Related Marker Compounds

Crude extract powder of *I. indigotica* was obtained from Sun Ten Pharmaceutical Co., Ltd., a Taiwanese manufacturer of concentrated herbal extracts. For each extract tested, 1 g of powder was dissolved in 40 mL ethyl acetate or methanol, then gently shaken overnight at room temperature. Extract solutions were collected following centrifugation at 12,000 rpm for 20 min, filtered using a Whatman number 1 filter paper, then lyophilized using in a freeze dryer (IWAKI FDR-50P). Each lyophilized extract powder was kept in sterile bottles at −20°C. Stock extract solutions (1 mg/mL) were dissolved in phosphate-buffered saline (PBS), sterilized using a 0.44 *μ*m syringe filter and stored at −80°C until used. Marker compounds of *I. indigotica* like adenosine, betulin, indigo, indirubin, tryptanthrin, lupeol, and 2-benzoxazolinone were purchased from Sigma Chemical Co. (St. Louis, MO.). Stock solution of marker compounds (20 mg/mL) was dissolved in dimethyl sulfoxide (DMSO), diluted with PBS. DMSO (0.005%, 0.05%, 0.5%, and 5%) was tested as solvent control.

### 2.3. Cell Viability Assay

To calculate cytotoxicity to BHK-21 cells and human promonocytic cells, cells were cultured overnight on 96-well plates. Medium containing DMSO (0.005, 0.05, 0.5, or 5%), *I. indigotica* extracts or marker compounds (0 *μ*g/mL, 0.1 *μ*g/mL, 1 *μ*g/mL, 10 *μ*g/mL, and 100 *μ*g/mL) were added and incubated for another 48 hours. Living cells and total HL-CZ cell count with(out) treatment were measured by staining with 0.4% trypan blue; viability was estimated as ratio of living/total cell counts. Quadruplicate wells were analyzed for each concentration. Cytotoxic concentration showing 50% toxic effect (CC_50_) was derived by computer program (provided by John Spouge, National Center for Biotechnology Information, National Institutes of Health).

### 2.4. Quantitative Assay of Virus Yields Using Plaque Assay

To test inhibitory effect of *I. indigotica* on JEV yields in human promonocytic cells, HL-CZ cells infected with JEV at multiplicity of infection of 0.5 and treated with DMSO (0.005, 0.05, or 0.5%), *I. indigotica* extract (1, 10, and 100 *μ*g/mL) or marker compound (0.1, 1, 10 *μ*g/mL) at the same time. At 24 and 48 h after inoculation, cultured supernatant from (un)treated JEV-infected cells was collected for measuring virus yields by plaque assay. A 10-fold serial dilution of cultured medium was added into the well of BHK-21 cell monolayer at 37°C for 1 h and overlaid with MEM medium containing 1.1% methylcellulose. Viral plaques were stained with naphthol blue-black dye after three-day incubation.

### 2.5. Plaque Reduction and Time-of-Addition Assay

To gauge inhibitory effect of *I. indigotica* by time of addition on JEV replication* in vitro*, pretreatment (prior to infection), simultaneous treatment (at the same time as infection), and posttreatment (after entry) experiments were performed. For the pretreatment experiment, BHK-21 cell monolayer was pretreated with/without various DMSO concentrations (0, 0.005, 0.05, or 0.5%), *I. indigotica* extract (0, 1, 10, and 100 *μ*g/mL) or marker compound (0, 0.1, 1, 10 *μ*g/mL) 1-h before infection. BHK-21 cell monolayer was overlaid with MEM medium containing 1.1% methylcellulose 1 h after infection, viral plaques stained with naphthol blue-black dye after three-day incubation. For simultaneous treatment, medium with/without various DMSO concentrations (0, 0.005, 0.05, or 0.5%), *I. indigotica* extract (0, 1, 10, and 100 *μ*g/mL) or marker compound (0, 0.1, 1, 10 *μ*g/mL) was mixed along with JEV at 100 pfu, then forthwith added into the well of BHK-21 cell monolayer at 37°C for 1 h and overlaid with MEM medium containing 1.1% methylcellulose for viral plaque assays. In posttreatment assay, BHK-21 cell monolayer was infected with JEV at 100 pfu for 1 h, followed by 1 h incubation with drug solutions and overlaid with MEM medium containing 1.1% methylcellulose, as described in plaque assay. Data represent means ± SD of three independent experiments. Inhibitory concentration showing 50% JEV plaque reduction (IC_50_) was determined by computer program (John Spouge, National Center for Biotechnology Information, National Institutes of Health).

### 2.6. Virus Attachment Assay

JEV (120 pfu) was mixed with medium containing various concentrations of DMSO (0, 0.005, 0.05, or 0.5%), *I. indigotica* extract (0, 1, 10, and 100 *μ*g/mL), or marker compound (0, 0.1, 1, and 10 *μ*g/mL), then immediately incubated with BHK-21 cell monolayer at 4°C to allow attachment. After 1 h incubation, each extract/virus or compound/virus mixture was removed, cell monolayer washed with cold PBS and overlaid with MEM medium containing 1.1% methylcellulose. After 3-day incubation at 37°C in a 5% CO_2_ incubator, plaques were stained, as described in plaque assay.

### 2.7. Virucidal Activity Assay

Virucidal assay was based on prior reports [14, 15]. JEV (10^5^ pfu) was mixed with medium containing DMSO (0.005%, 0.05%, and 0.5%), *I. indigotica* extract (1, 10, and 100 *μ*g/mL) or marker compound (0.1, 1, and 10 *μ*g/mL) and incubated for 60 min at 4°C. A 1000-fold dilution of each extract/virus or compound/virus mixture was added onto BHK-21 cell monolayer in 6-well plates. After 1 h incubation, mixtures were removed and washed with PBS, while monolayer was overlaid with MEM medium containing 1.1% methylcellulose; residual infectivity and inhibitory concentration showing 50% JEV plaque reduction (IC_50_) were determined, all as described in plaque assay.

### 2.8. Mouse Protection Assay

Groups (*n* = 10) of 2-week-old BALB/c mice were intracerebrally infected with 1 × 10^5^ pfu of virulent JEV strain Beijing-1 then underwent three intracerebral treatments with 30 *μ*g/100 *μ*L of *I. indigotica* extract or marker compound (1 mg/kg of body weight) using 100 *μ*L syringes at 2, 24, and 48 h after infection. Two additional groups were infected with JEV and received PBS or DMSO (0.05%) treatment as solvent controls. Survival rates were monitored every day for one week.

### 2.9. Statistical Analysis

ANOVA using SPSS program (version 10.1, SPSS Inc., IL, USA) or Student *t*-test analyzed data, *P* value less than 0.05 considered statistically significant.

## 3. Results

### 3.1. Cytotoxicity of *I. indigotica* Extracts and Related Marker Compounds

To test cytotoxicity, BHK-21 and HL-CZ cells were treated with both *I. indigotica* extract and related marker compounds at concentrations of 0.1–1000 *μ*g/mL. Since diluted solutions of indigo and indirubin contained 0.0005% 0.005%, 0.05%, 0.5%, and 5% DMSO, cells were also treated with serial dilution of DMSO as solvent control. Cytotoxicity assay indicated extracts of *I. indigotica* by ethyl acetate and methanol less toxic to BHK-21 kidney cells (CC_50 _≧ 100 *μ*g/mL) than human promonocytic HL-CZ cells (CC_50_ = 49.02 *μ*g/mL). In solvent controls, both cell types had maximum DMSO tolerance under 0.5%; viability of indigo- and indirubin-treated cells was gauged at concentrations of 0.1–100 *μ*g/mL containing less than 0.5% DMSO. CC_50_ values of indigo and indirubin varied from 26.88 *μ*g/mL (BHK-21 cells treated with indigo) to 57.47 *μ*g/mL (BHK-21 cells treated with indirubin) (Table 1). Other *I. indigotica* related marker compounds like tryptanthrin, adenosine, betulin, lupeol, and 2-benzoxazolinone showed high toxicity to both cell lines (CC_50_ < 25 *μ*g/mL). Compared to ethyl acetate and methanol extracts, indigo and indirubin manifested low toxicity to such cells, being available for *in vitro* and *in vivo* activity against JEV.

### 3.2. Inhibition of JEV Yield by Indigo and Indirubin

**Figure 1:**
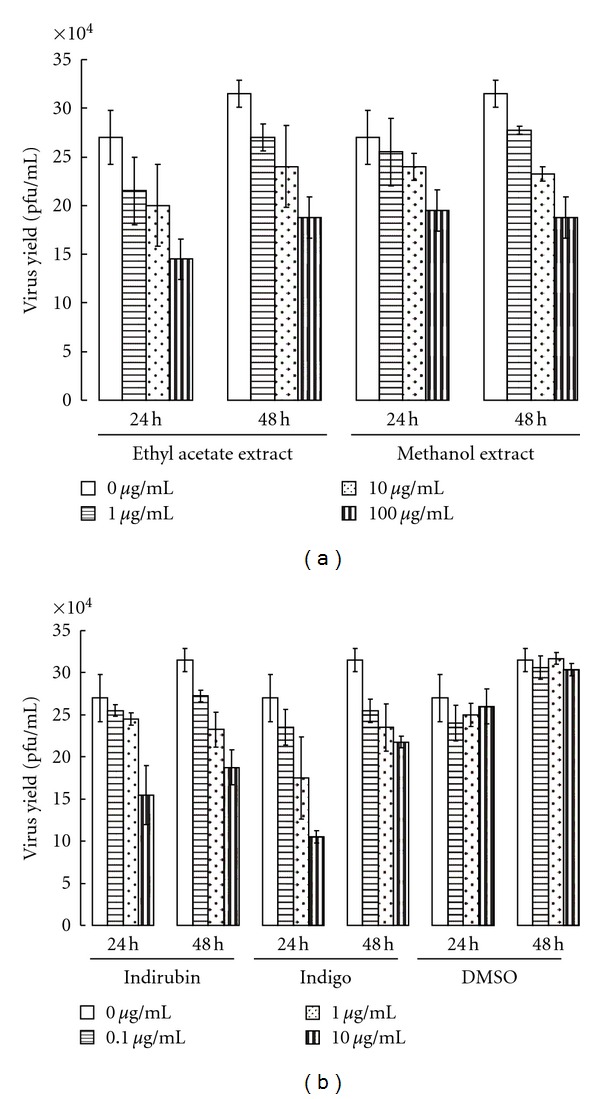
Inhibition of virus yield by *I. indigotica* extract, indigo and indirubin. HL-CZ cells were infected with JEV at a MOI of 0.5 at the same time as treatment with serial dilution of *I. indigotica* extracts (a) indigo and indirubin (b). Cultured supernatants in infected cells were harvested at 24 and 48 h after infection. Virus yield was performed as described in plaque assay.

To detect inhibition of virus yield in human promonocytic HL-CZ cells by *I. indigotica*, virus titers in cultured supernatants for JEV-infected HL-CZ cells with or without treatment were measured 24 and 48 hours after infection, using plaque assay (Figure 1). *I. indigotica* extract, indigo and indirubins showed dose-dependent inhibition of JEV replication in HL-CZ promonocytic cells, but no time-dependent inhibitory effect on JEV production *in vitro*. Particularly, indigo (10 *μ*g/mL) and indirubin (10 *μ*g/mL) showed virus yield reduced by approximately 40% after 24 h incubation.

### 3.3. Inhibition of JEV Replication by Pretreatment of *I. indigotica* Extracts, Indigo and Indirubin

**Figure 2:**
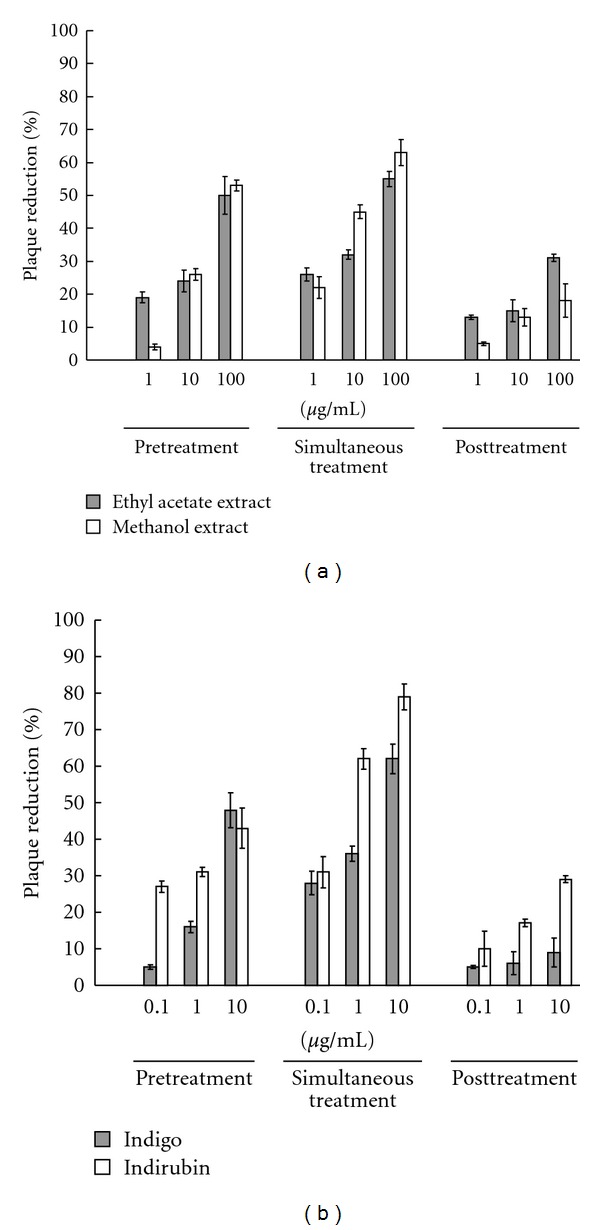
Plaque reduction of JEV by *I. indigotica* and its major components in pre-, simultaneous, and post-treatment assays. Serial dilutions of ethyl acetate and methanol extracts of *I. indigotica* (a) and its components indigo and indirubin (b) were pretreated before virus absorption, simultaneously with virus infection, or posttreated after virus absorption (JEV at 100 pfu/well). BHK-21 cell monolayer was incubated with virus/compound mixture at 37°C for 1 h, then overlaid with MEM medium containing 1.1% methylcellulose. Viral plaques were stained with naphthol blue-black dye after 3 days of incubation.

To ascertain time-of-addition effect of *I. indigotica *on JEV replication, BHK-21 cells were pretreated (prior to infection), simultaneously treated (at the same time as infection), or posttreated (after entry) with various concentrations of *I. indigotica* extracts, indigo and indirubin as well as serial dilution of DMSO (solvent control). With simultaneous treatment, both indigo and indirubin showed concentration-dependent inhibition of JEV plaques *in vitro*: IC_50_ plaque reduction values of 91.57 *μ*g/mL for ethyl acetate extract, 78.47 *μ*g/mL for methanol extract, 37.49 *μ*g/mL for indigo, and 13.68 *μ*g/mL for indirubin (Figure 2 and Table 2). Both indigo and indirubin tallied a therapeutic index (CC_50_/IC_50_) of >10. Meanwhile, each dilution of DMSO had no significant effect on plaque reduction (data not shown).

Both pre- and post-treatment plaque reduction assays tested antiviral effect of *I. indigotica *on JEV replication. In pretreatment assay, both indigo and indirubin pretreated before JEV adsorption showed antiviral activity similar to simultaneous treatment assay (Figure 2 and Table 2). However, posttreatment of *I. indigotica* extracts, indigo and indirubin was ineffective in antiviral activity after virus entry. Results demonstrated pretreatment of *I. indigotica* extracts, indigo and indirubin that affects JEV replication *in vitro*.

### 3.4. Inhibition of Virus Attachment by Indigo and Indirubin

**Figure 3:**
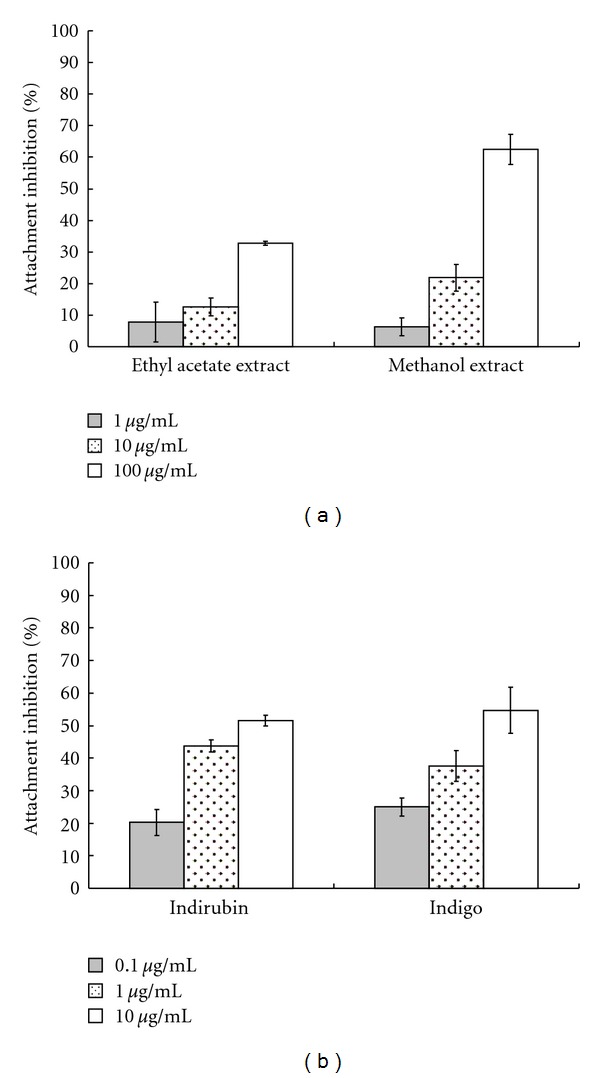
Inhibition of virus attachment by *I. indigotica* extract, indigo and indirubin. *I. indigotica* extracts (a) indigo and indirubin (b) were serially diluted and mixed with JEV (120 pfu), each mixture incubated 1 h with BHK-21 cell monolayers at 4°C. Then virus/compound mixture was removed and cell monolayer washed with cold PBS. Residual infectivity was performed as described in plaque assay.

To rate inhibitory effect of *I. indigotica* on virus attachment, JEV mixture (120 pfu) with *I. indigotica* extract, indigo, indirubin, or 0.5% DMSO (solvent control) was immediately incubated at 4°C with BHK-21 cell monolayer to allow attachment alone. After virus attachment at 4°C for 1 h, each mixture was removed and cell monolayer washed with PBS. Residual infectivity derived by plaque assay yielded IC_50_ values of methanol extracts, indigo and indirubin below pre- and simultaneous treatment (Figure 3, Table 2). However, 0.5% DMSO had no significant effect on virus attachment (data not shown). These results demonstrated a potent inhibitory effect of methanol extracts, indigo and indirubin on JEV attachment.

### 3.5. Virucidal Activity of Indigo and Indirubin

**Figure 4:**
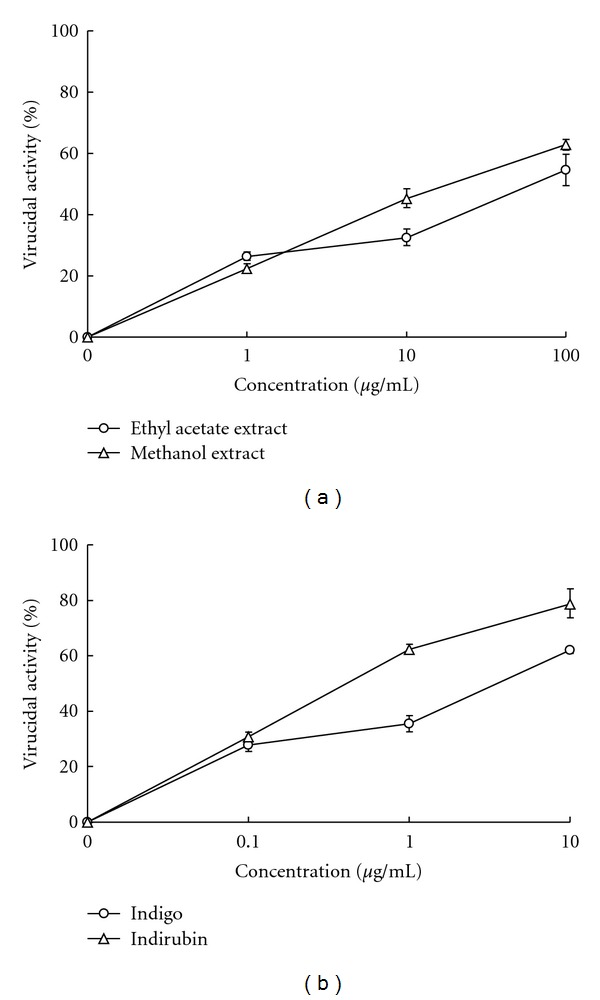
Virucidal activities of *I. indigotica* and its major components. *I. indigotica* extracts (a) indigo and indirubin (b) were serially diluted and mixed with JEV. Each virus/compound mixture was incubated at 4°C for 1 h, then added onto the BHK-21 cell monolayer at 37°C for another 1 h. The virus/compound mixture was removed from 6-well plates and cell monolayer washed with PBS. Residual infectivity was performed as described in plaque assay.

To ascertain whether* I. indigotica* has a virucidal action by directly interfering with virus particles, JEV was preincubated with both indigo and indirubin at 4°C for 1 h, and residual infectivity tested by plaque assay (Figure 4 and Table 2). Both indigo and indirubin exhibited concentration-dependent virucidal activity as well as significant inhibitory effect on residual infectivity compared to controls. Virucidal IC_50_ values against JEV were 65.79 *μ*g/mL of ethyl acetate extract, 22.17 *μ*g/mL of methanol extract, 3.03 *μ*g/mL of indigo, and 0.47 *μ*g/mL of indirubin. Moreover, virucidal IC_50_ values were below pre-, simultaneous, and posttreatment as well virus attachment assay (Table 2), revealing that *I. indigotica* directly inactivated JEV particles, exhibiting a potently virucidal action.

### 3.6. Protection against Lethal Challenge in Mice by Indigo and Indirubin

**Figure 5:**
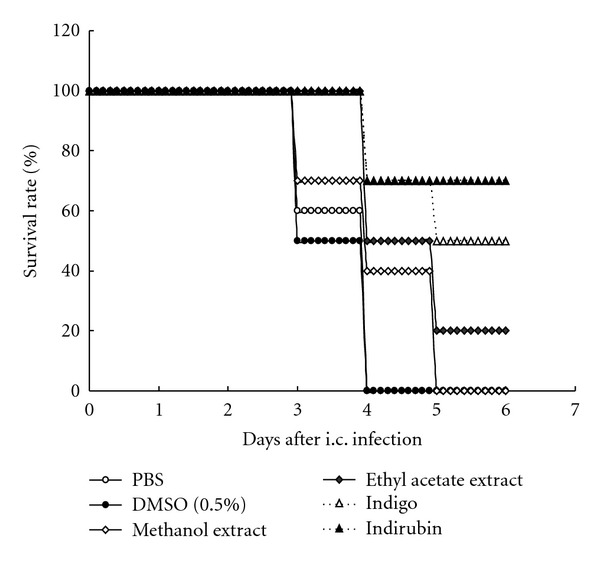
Mouse protection against lethal JEV challenge by *I. indigotica*. Groups of 2-week-old BALB/c mice were intracerebrally infected with JEV at 1 × 10^5^ pfu, then underwent three intracerebral treatments with *I. indigotica* extract, indigo, or indirubin (1 mg/kg of body weight) at 2, 24, and 48 h after infection. Two additional groups were infected with JEV and received PBS or DMSO (0.05%) treatment as solvent controls. Survival rates were monitored daily.

To investigate* in vivo* protective potential of *I. indigotica*, groups of mice were intracerebrally challenged with lethal dose of virulent JEV strain Beijing-1 and treated with extracts, indigo, indirubin, PBS, or 0.5% DMSO at 2, 24, and 48 h after infection. Survival rate of the indirubin-treated group on Day 6 after infection was 70%, starkly higher than others: for example, indigo- (50%) and ethyl acetate extract-treated (20%) (Figure 5). None in the methanol extract-, PBS-, or DMSO-treated groups survived, indicating indirubin as superior to indigo, ethyl acetate extract better than methanol extract in mouse protection against lethal i.c. challenge with JEV.

## 4. Discussion

This study demonstrated *I. indigotica* extracts as having low cytotoxicity and concentration-dependent inhibitory effects on JEV replication *in vitro*: for example, reducing virus yield, blocking virus attachment, and virucidal activity (Figures 1–4, Tables 1 and 2). *I. indigotica* extract displays multiple antiviral and immunomodulatory activity against foot-and-mouth disease, rabies, HIV-1, influenza A, and SARS-coronavirus [1, 3–9]. Our results indicate antiviral potential of *I. indigotica* against JEV.

Among related compounds, indirubin manifested potent anti-JEV activities with plaque reduction (IC_50_ = 13.68 *μ*g/mL via simultaneous treatment), virus attachment inhibition (IC_50_ = 5.10 *μ*g/mL) and virucidal inactivation (IC_50_ = 0.47 *μ*g/mL) (Figures 2–4 and Table 2). Indirubin also concentration-dependently reduced virus yield in cell cultures (Figure 1(b)). Indigo effectively inhibited JEV replication *in vitro*, reduced virus yield and attachment (Figures 1(b) and 3(b)), showing greater virucidal activity (IC_50_ = 3.03 *μ*g/mL) than *I. indigotica* extracts. Indirubin and indigo had a potent virucidal activity through directly inactivating virus particles, linking with a better inhibition of JEV replication by pretreatment, and a significant reduction of virus attachment and yield *in vitro*. Similar antiviral effect of indirubin against pseudorabies virus has been also reported [5]. The inconsistency in anti-JEV abilities among reducing virus yield, virus attachment and virucidal activity could be due to the possibility that cells rapidly uptakes indirubin and indigo, then metabolizes them as inactive production, being supported in a prior report [16].

Indirubin had potent *in vivo* protection against intracerebral JEV challenge at lethal dose, more than indigo, ethyl acetate extract, or methanol extract (Figure 5). Indirubin likewise regulates immunomodulatory activity on RANTES expression in influenza-infected bronchial epithelial cells [8], showing broad-spectrum antiviral activity and more effective virucidal action against JEV. In sum, *I. indigotica* contains potential antiviral components against JEV, and so forth through virucidal actions. Among major *I. indigotica* components, indirubin manifests potential for antiviral activity against JEV infection, which could yield new anti-JEV agents.

## Figures and Tables

**Table 1:** Cytotoxic effect of *I. indigotica* extracts and its marker components on BHK-21 and HL-CZ cells.

*Isatis indigotica*	CC_50_ (*μ*g/mL)	
	BHK-21^a^	HL-CZ^a^
Ethyl acetate extract	>100	49.02 ± 2.04
Methanol extract	>100	>100
Indigo	86.88 ± 3.72	99.41 ± 0.54
Indirubin	>100	64.89 ± 1.08
Tryptathrin	4.57 ± 0.26	1.74 ± 0.34
Adenosine	24.10 ± 2.57	15.38 ± 1.05
Betulin	13.30 ± 2.58	2.10 ± 0.78
Lupeol	9.53 ± 1.32	3.44 ± 1.17
2-Benzoxazolinone	19.92 ± 1.57	67.33 ± 10.2

^a^Measured using trypan blue staining.

**Table 2:** Antiviral effect of *I. indigotica* extracts and its marker components against JEV.

*Isatis indigotica*	IC_50_ (*μ*g/mL) of pretreatment	IC_50_ (*μ*g/mL) of simultaneous treatment	IC_50_ (*μ*g/mL) of posttreatment	IC_50_ (*μ*g/mL) of virus attachment	IC_50_ (*μ*g/mL) of virucidal assay
Ethyl acetate extract	138.81 ± 3.71	91.57 ± 3.54	>200	>200	65.79 ± 3.54
Methanol extract	85.46 ± 8.54	78.47 ± 3.06	>200	50.57 ± 2.12	22.17 ± 3.06
Indigo	11.79 ± 0.10	37.49 ± 3.18	>50	5.15 ± 0.18	3.03 ± 3.18
Indirubin	23.50 ± 0.89	13.68 ± 2.83	>50	5.10 ± 1.32	0.47 ± 2.83
